# 1210. Prospective review of provider discontinuation of inappropriate intravenous (IV) vancomycin at a community teaching hospital in Chicago

**DOI:** 10.1093/ofid/ofad500.1050

**Published:** 2023-11-27

**Authors:** Jennifer Oderinde, Anthony Chiang, Alicia Juska, O L U W A D A M I L O L A A ADEYEMI

**Affiliations:** Swedish Hospital NorthShore University HealthSystem, Chicago, Illinois; Advocate Lutheran General Hospital, Evanston, IL; Swedish Hospital NorthShore University HealthSystem, Chicago, Illinois; Swedish Hospital Part of NorthShore University HealthSystem, Chicago, Illinois

## Abstract

**Background:**

Vancomycin is often started empirically for its reliability with gram-positive pathogens, particularly *Methicillin Resistant Staph aureus* (MRSA). Consequently, it is often over-prescribed. Antimicrobial stewardship programs (ASP) have used time-outs to ensure therapies are tailored to the patient with proper indication and coverage.

**Figure:**
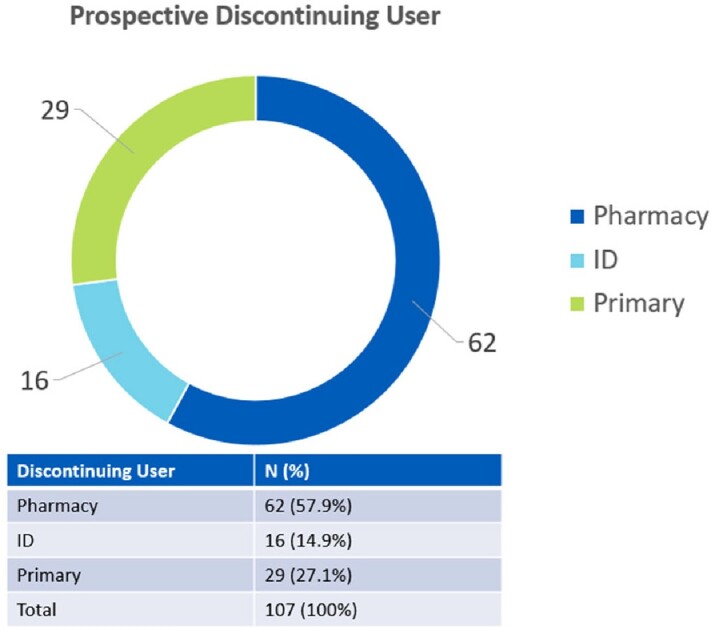

**Methods:**

Between November 2022 and February 2023 provider discontinuation of IV vancomycin was prospectively reviewed when it was no longer needed at a 298 licensed bed community teaching hospital in Chicago. Results of culture sensitivities including MRSA nares screen, blood, and wound cultures were used to analyze appropriateness. Appropriateness was defined as having a positive MRSA culture, positive MRSA nares for treatment of PNA only, gram positive infections with a true and severe beta-lactam allergy, gram positive infections with beta-lactam resistant pathogens (i.e. ampicillin resistant *Enterococcus*), purulent cellulitis without culture data, and pending or negative cultures for deep-seated infections (i.e. CNS, Cardiovascular, BJI) with ID consult.

**Results:**

153 patients met criteria for discontinuation of IV vancomycin and 107 of those were able to be discontinued. Inappropriate regimens were continued in 46 patients for various indications including pneumonia, skin and soft tissue infections, intra-abdominal infections, urinary tract infections, and endocarditis without infectious disease (ID) consult.

Of the 107 patient encounters, pharmacists intervened and discontinued 62 (57.9%), ID discontinued 16 (14.9%) and the primary medical team discontinued 29 (27.1%).

**Conclusion:**

30% (46/153) of patients studied continued an inappropriate vancomycin regimen. There is room for improvement in regards to proper education surrounding antimicrobial stewardship. An antimicrobial time-out can help further decrease inappropriate vancomycin use.

**Disclosures:**

**All Authors**: No reported disclosures

